# Comparison of pregnancy outcomes between 4th day morula and 5th day blastocyst after embryo transfer: a retrospective cohort study

**DOI:** 10.1186/s12884-024-06597-7

**Published:** 2024-07-03

**Authors:** Yiqun Sun, Qi Shen, Haitao Xi, Liucai Sui, Yanghua Fu, Junzhao Zhao

**Affiliations:** Department of Obstetrics and Gynecology, Reproduction Center, the Second Affiliated Hospital and Yuying, Children’s Hospital of Wenzhou Medical University, Wenzhou, 325000 China

**Keywords:** Early follicular phase prolonged protocol, Morula, Blastocyst, Assisted reproduction, IVF/ICSI outcome

## Abstract

**Background:**

This study was designed to evaluate pregnancy outcomes between morulae transferred on day 4 (D4) and blastocysts transferred on day 5 (D5).

**Methods:**

From September 2017 to September 2020, 1963 fresh transfer cycles underwent early follicular phase extra-long protocol for assisted conception in our fertility center were divided into D4 (324 cases) and D5 (1639 cases) groups, and the general situation and other differences of patients in both groups were compared. To compare the differences in pregnancy outcomes, the D4 and D5 groups were further divided into groups A and B based on single and double embryo transfers. Furthermore, the cohort was divided into two groups: those with live births (1116 cases) and those without (847 cases), enabling a deeper evaluation of the effects of D4 or D5 transplantation on assisted reproductive outcomes.

**Results:**

In single embryo transfer, there was no significant difference between groups D4A and D5A (*P* > 0.05). In double embryo transfer, group D4B had a lower newborn birthweight and a larger proportion of low birthweight infants (*P* < 0.05). The preterm delivery rate, twin delivery rate, cesarean delivery rate, and percentage of low birthweight infants were lower in the D5A group than in the D5B group (*P* < 0.05). Analysis of factors influencing live birth outcomes further confirmed the absence of a significant difference between D4 and D5 transplantation in achieving live birth (*P* > 0.05).

**Conclusion:**

When factors such as working life and hospital holidays are being considered, D4 morula transfer may be a good alternative to D5 blastocyst transfer. Given the in vitro fertilization/intracytoplasmic sperm injection (IVF/ICSI) success rate and risk of twin pregnancy, D4 morula transfer requires an adapted decision between single and double embryo transfer, although a single blastocyst transfer is recommended for the D5 transfer in order to decrease the twin pregnancy rate. In addition, age, endometrial thickness and other factors need to be taken into account to personalize the IVF program and optimize pregnancy outcomes.

**Supplementary Information:**

The online version contains supplementary material available at 10.1186/s12884-024-06597-7.

## Introduction

 The advancement of Assisted Reproductive Technology (ART), including the improvement of embryo culture media and the optimization of culture techniques, has enabled the development of embryos into blastocysts in vitro [1]. Numerous studies have demonstrated that compared to cleavage stage embryo, blastocysts have greater implantation and developmental potential [2–4]. As a result, many centers have shifted their transfer strategy from cleavage embryo transfers on day 3 to blastocyst transfers on day 5. Due to the lack of typical morphological markers [5], morulae on day 4 have long been understudied in humans, and the corresponding value of morula transfers has frequently been overlooked. However, it has recently been demonstrated that the densification process at the morula stage involves multiple self-correcting mechanisms that may be critical in determining embryo quality [6], as well as being critical for blastocyst formation, establishment of the first cell lineage, and the entire developmental process [7, 8], which means that morula transfer may also be a feasible transfer strategy. In addition, studies have shown that the use of ART techniques, particularly dual embryo transfer, significantly increases the rate of twin pregnancies [9], which can increase the risk of pregnancy and seriously endanger the mother’s and child’s life and health [10]. To reduce the number of twin pregnancies while successfully assisting pregnancy, our center prefers to use a single blastocyst transfer strategy. However, many patients choose D4 morula transfer due to time constraints such as work life and hospital holiday breaks. And some patients hold the belief that transferring two embryos can enhance the success rate of IVF and request the transfer of two embryos. It should be added that blastocyst culture is more complicated and delicate compared to morula culture, which requires strict laboratory environment and technical support [11, 12]. On the other hand, the cultivation of morula and cleavage embryo is relatively simple, requires less laboratory environment and technical support. Some clinics, due to technical constraints, may prefer D4 morula or D3 cleavage embryo for transfer. In this study, we compared the pregnancy outcomes of D4 morula and D5 blastocyst, single embryo and double embryos using the early follicular stage extra-long protocol, and investigated the benefits and drawbacks of various transfer strategies in order to provide a foundation for individualized transfer protocols.

## Materials and methods

### Research subject

**Fig. 1:**
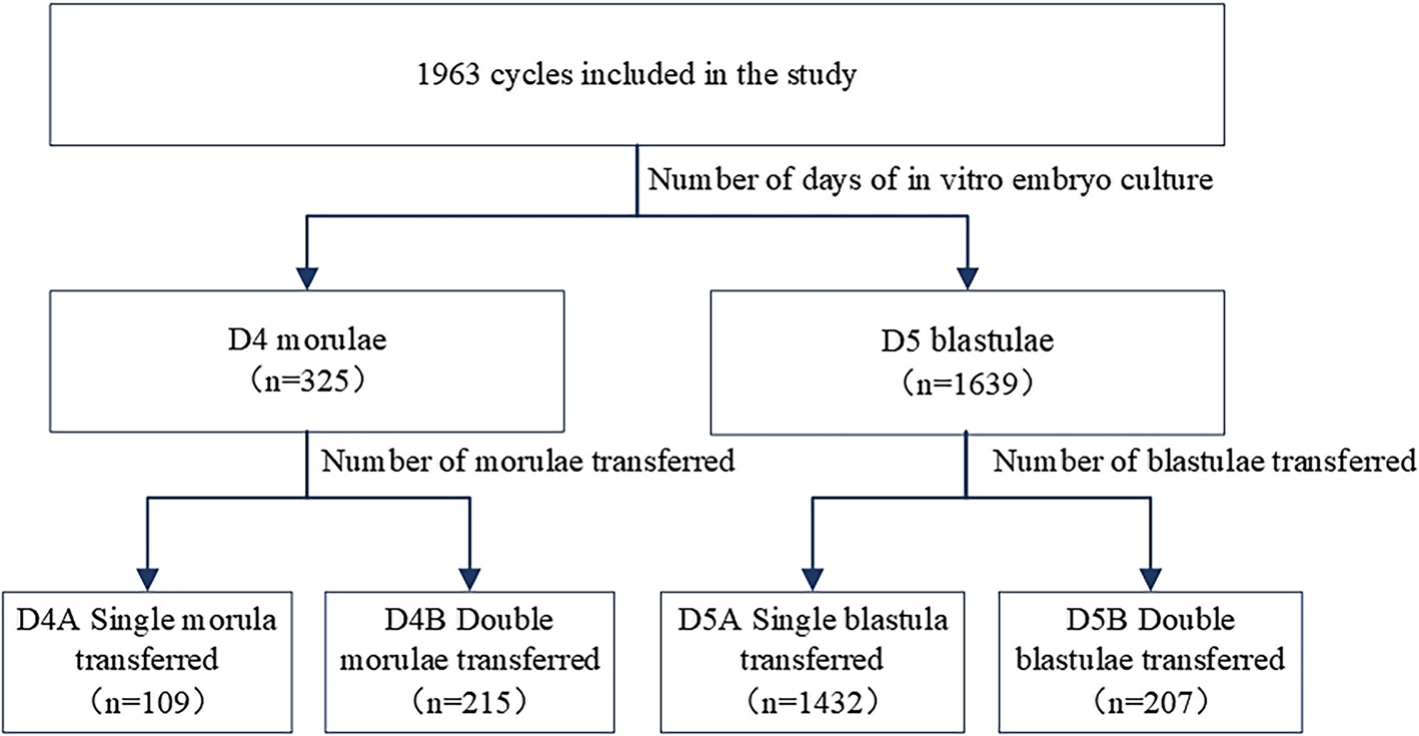
Grouping of morulae on day 4 and blastocysts on day 5 after fertilization using an early follicular phase prolonged protocol. D4, the fourth day after fertilization; D5, the fifth day after fertilization; D4A, single morula was transplanted; D4B, double morulae were transplanted; D5A, single blastocyst was transplanted; D5B, double blastocysts were transplanted

Retrospective analysis of 1963 cycles undergoing early follicular phase ultra-long protocol assisted conception at our fertility center from September 2017 to September 2020, divided into D4 (324 cases) and D5 (1639 cases) groups based on the number of days of in vitro culture after fertilization, and further divided D4 and D5 into groups A and B based on single and double embryo transfer to compare their outcomes, as show in Fig. 1. To compare D4A with D4B, D5A with D5B, D4A with D5A, D4B with D5B, and to clarify the differences in pregnancy outcomes between D4 morula and D5 blastocyst, as well as single embryos and double embryos for transfer. The 1963 cycles were divided into a live birth group (1116 cases) and a non-live birth group (847 cases) according to whether a live birth outcome was obtained, and the effect of D4 or D5 transplantation on live birth outcome was further verified using univariate analysis and Logistic regression analysis. Inclusion criteria: 1. patient age between 20 and 42 years; 2. early follicular phase ultra-long protocol was used to assist pregnancy; 3. number of day3(D3) high-quality embryos ≥ 3. Exclusion criteria:1. uterine pathologies such as adenomyosis, submucous myomas, severe uterine adhesions, uterine malformations; 2. history of adverse pregnancy such as recurrent miscarriage, stillbirth and multiple inductions; 3. chromosomal abnormalities.

### Assisted reproductive process

To limit the impact of diverse IVF regimens on pregnancy outcomes, all cycles in this study were completed using the early follicular phase extra-long regimen, which was routinely utilized to facilitate pregnancy in our center. On days 2–5 of menstruation, 3.75 mg of gonadotropin releasing hormone agonist (GnRH-a) was administered intramuscularly to suppress pituitary function. After 28–38 days, an ultrasound and endocrine examination would be conducted. When endometrial thickness was less than 5 mm, follicle stimulating hormone (FSH) was less than 5mIU/ml, luteinizing hormone (LH) was less than 5mIU/ml, and estradiol(E2) was less than 50pg/ml, the pituitary gland was considered completely downregulated. At this time, gonadotropin (Gn) would be used to initiate ovulation, and the type and dosage of Gn would be adjusted continuously based on ultrasound and serum sex hormone level. An intramuscular injection of 4000–10,000 IU of chorionic gonadotropin was given when at least one follicle measures at least 20 mm in diameter or when two follicles measure at least 18 mm in diameter. Oocytes were carried out under vaginal ultrasonography guidance 36 h later, and embryos were cultivated after in vitro fertilization of the oocytes, embryo transfer was performed under abdominal ultrasound guidance. Serum human chorionic gonadotropin (hCG) levels were examined two weeks after transfer to see if there was a pregnancy. If the hCG level was positive, an ultrasound two weeks later revealed an intrauterine gestational sac, indicating a clinical pregnancy. Follow up patients at 12 weeks, 24 weeks, 36 weeks, and 2 weeks postpartum regarding miscarriage, premature birth, live birth, and other related conditions.

### Embryo culture and classification

The quality of embryos was closely related to clinical outcomes [13, 14], and we had strict requirements for embryo quality in order to control the interference of different embryo quality on study outcomes.

**Fig. 2:**
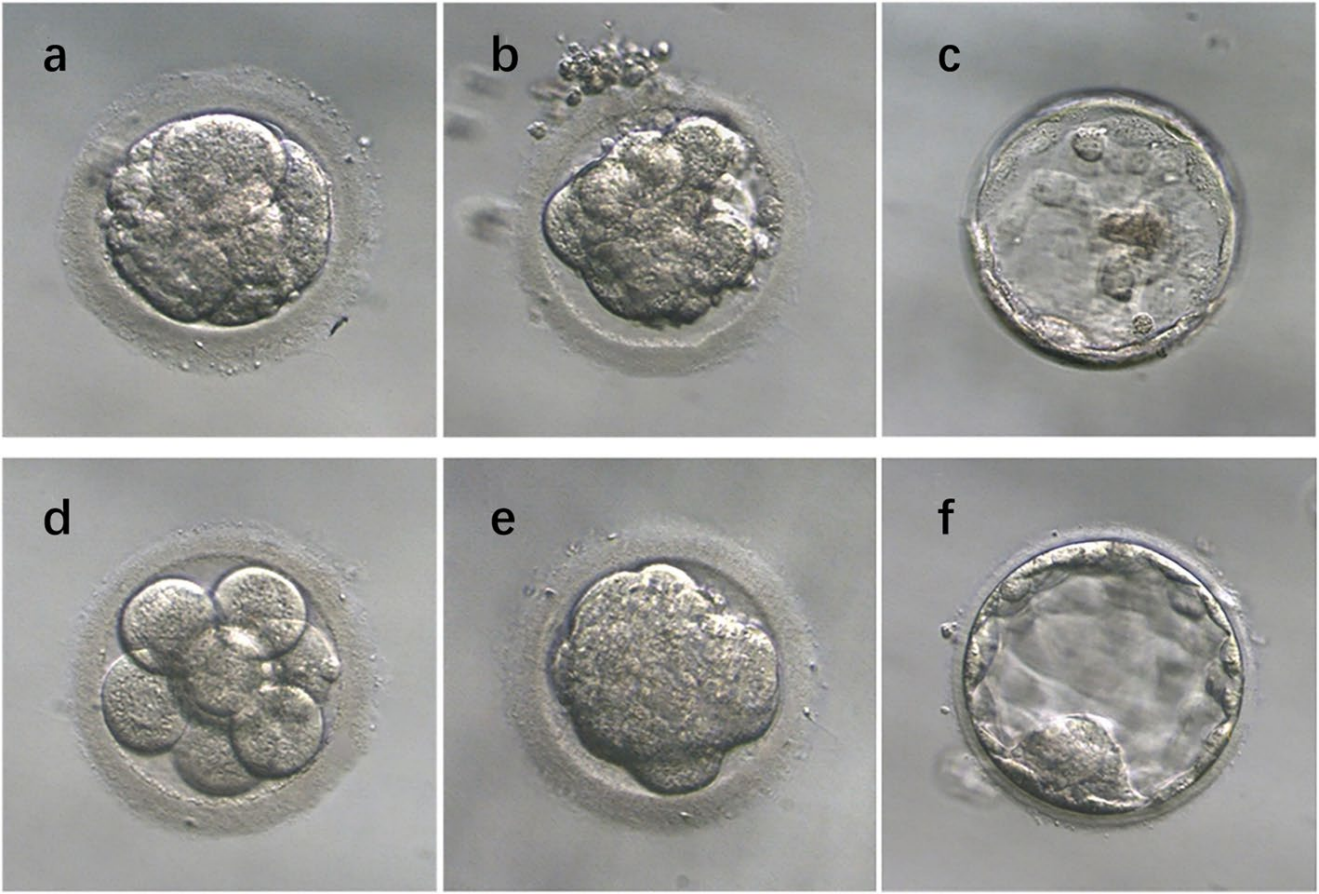
Photomicrographs of different grades of D3 cleavage embryo, D4 morula, and D5 blastocyst. **a** Inferior D3 cleavage embryo. The embryo had uneven surfaces, irregular shapes, and was filled with more fragments and visible cell granules. **b** Inferior D4 morula. Only some blastomeres underwent compaction, that was partial compaction, and other cells remained in a separated status, then a smaller compacted multicellular mass would form. **c** Inferior D5 blastocyst. The blastocyst cavity was not significantly dilated, the number of ICM cells was low, and the TE cells were sparse. **d** High quality D3 cleavage embryo. The embryo was of uniform size, regular shape, full hyaline band, uniform cell quality, no granularity and few fragments. **e** High quality D4 morula. The embryo was nearly fully compacted with all blastomeres undergoing the compact process, and a large multicellular mass was expected. **f** High quality D5 blastocyst. The blastocyst cavity was significantly enlarged in volume, the zona pellucid was thinned, the number of ICM cells was high and tightly arranged, and the number of TE cells was high, forming a tight epithelium. Original magnification: x200.

In this study, we conducted sequential embryo culture, during which embryos were placed in media that mirrored the in vivo environment suitable for their respective developmental stages. The media utilized were from the G-Series, specifically G1 and G2 (supplied by Vitrolife, Sweden). Initially, oocytes were fertilized in G-IVF plus medium, and subsequently, the cleavage-stage embryos were incubated in G1 medium, maintained at 37 °C, 6% CO2, and saturated humidity. On Day 3, selected cycles containing at least three high-quality embryos were divided into D4 and D5 groups. These embryos were then transferred to G2 medium for blastocyst development, cultured under conditions of 37 °C, 6% CO2, 5% O2, and saturated humidity. The culture process continued until Day 4 or Day 5, and ultimately, we chose 1–2 embryos with the utmost quality on either Day 4 or Day 5 for transplantation. According to the Istanbul consensus [15], the embryo quality was graded according to the size, uniformity and amount of nucleus-free fragments of the D3 oocyte cleavage spheres. A high-quality embryo should have 6–10 cells, uniform blastomere size, few fragments, and no multinucleation on D3 after fertilization. High-quality embryos included 7 A, 8 A, 9 A, 7B, 8B, and 9B.The agreement on Day 4 was that an optimum embryo at this stage would be compacted or compacting, the compaction should include virtually all the embryo volume and would have entered a fourth round of cleavage [16]. If more than half of the embryos are expelled, this may be associated with a poor prognosis [17]. Blastocyst scoring used the Gardner scale [18, 19], which scored the degree of blastocyst expansion, inner cell mass, and trophectoderm cell development, respectively. Blastocysts with tightly arranged cells, almost free of debris and not yet hatched were usually considered as the most suitable blastocysts for transfer, including 3AA/AB/BA/BB, 4AA/AB/BA/BB, and 5AA/AB/BA/BB, as shown in Fig. 2. Patients who had one or more excellent embryos transferred were considered excellent embryo transfers, and those who had only one or two non- excellent embryos transplanted were considered non- excellent embryo transfers. The remaining transferable embryos were frozen, and the number and quality of transferred embryos, blastocysts raised, and frozen embryos were all counted.

### Observation indicators

Each group was examined for demographic characteristics, clinical and laboratory outcomes, pregnancy outcomes and other indicators. The primary outcome indicators were the clinical pregnancy rate (number of clinical pregnancy cycles/number of transplantation cycles×100%), miscarriage rate (number of miscarriage cycles/number of clinical pregnancy cycles×100%), and live birth rate (number of live births/number of transplantation cycles×100%) [20]. Secondary outcome indicators were the preterm birth rate, natural birth rate, cesarean delivery rate, neonatal weight, and other observations. As a major complication of ovulation promotion, we also compared the incidence of ovarian hyperstimulation syndrome (OHSS) in each group.

### Statistics analysis

For statistical analysis, SPSS 25.0 software (IBM Corp, Armonk, NY, USA) was utilized. The data was examined for normality. The average value of normally distributed measures was expressed as the mean ± standard deviation, and the T-Test for two independent samples was used to compare groups. While non-normally distributed measures were expressed as M (P25, P75), and the Wilcoxon rank sum test was used to compare groups. Count data was reported as percentages, and group comparisons were performed using the Chi-Square Test or Fisher-Yates-Test. A one-way analysis of variance (ANOVA) was performed with live birth outcome as the dependent variable, and the factors affecting the live birth rate were analyzed by including the variance items, the number of days to transfer, and the number of embryos transferred in a binary Logistic regression. The receiver operating characteristic curve (ROC curve) was plotted, and the area under the curve (AUC) was calculated; the optimal cut-off value was the maximum value of the Youden index. *P* values < 0.05 were considered significant.

## Results

### General information

Among 1963 cycles, we transferred morulae in 324 cycles and blastocysts in 1639 cycles. Table 1 summarizes the demographic characteristics of the D4 and D5 groups. There were no significant differences between these two groups in terms of age, Body mass index (BMI), years of infertility, Anti-Müllerian Hormone (AMH), basal FSH, basal LH or etiology of infertility. As shown in Table 2, with respect to clinical and laboratory outcomes, there were no differences in LH, E2, Progesterone(P) after downregulation, and LH, P on hCG triggering day, excellent embryo transfer rate, normal fertilization rate and quality embryo rate. However, the basal E2, E2 and endometrial thickness at hCG triggering, and the number of retrieved oocytes, fertilized oocytes, D3 embryos, and quality D3 embryos, number of blastocysts raised, number of quality blastocysts, number of embryos frozen, blastocyst rate, quality blastocyst rate were lower in the D4 group than in the D5 group, while the percentage of single embryo transfer was higher in the D5 group than in the D4 group. Because single and double embryo transfer rates differed between D4 and D5, we further separated D4 and D5 into D4A, D4B, D5A, D5B according to the number of embryos transferred.

**Table 1:** Comparison of demographic characteristics in two groups

Variables	D4 group(*n* = 324)	D5 group(*n* = 1639)	*P* value	
Age (years)	31.36 ± 4.42	31.18 ± 4.27		0.487
BMI (kg/m2)	21.79 ± 3.19	21.96 ± 3.12		0.356
Duration of infertility (years)	3.34 ± 2.43	3.34 ± 2.39		0.978
AMH (ng/ml)	3.68 ± 3.10	4.26 ± 3.38		0.059
Basic FSH (mIU/mL)	7.99 ± 2.98	7.93 ± 4.89		0.864
Basci LH/(IU/L)	5.63 ± 7.75	6.90 ± 11.02		0.062
E2/(pg/mL)	48.63 ± 29.29	55.51 ± 59.20		0.020
Etiology of infertility (%)				
Tubal	47.53(154/324)	46.31(759/1639)		0.687
PCOS	10.80(35/324)	14.15(232/1639)		0.108
Endometriosis	2.47(8/324)	1.65(27/1639)		0.307
Ovarian	5.86(19/324)	4.09(67/1639)		0.153
Combined	11.73(38/324)	11.65(191/1639)		0.969
Other	21.60(70/324)	22.15(363/1639)		0.830

*BMI *Body mass index, *AMH *Anti-Müllerian Hormone, *FSH* Follicle stimulating hormone, *LH* Luteinizing hormone, *E2* serum estradiol, *PCOS *Polycystic ovary syndrome

**Table 2:** Comparison of clinical and laboratory outcomes between the two groups

Variables	D4 group(*n* = 324)	D5 group(*n* = 1639)	*P* value
LH after deregulation (IU/L)	0.44 ± 0.39	0.55 ± 2.23	0.405
E2 after deregulation (pg/ml)	24.48 ± 12.82	27.83 ± 121.13	0.639
P after deregulation (nmol/L)	0.51 ± 0.42	0.56 ± 2.11	0.639
LH on trigger day (IU/L)	0.73 ± 0.56	0.72 ± 0.64	0.830
E2 on trigger day (pg/ml)	1833.07 ± 1023.23	2400.88 ± 1168.62	0.001
P on trigger day (nmol/L)	0.63 ± 0.32	1.44 ± 26.18	0.401
Endometrial thickness on trigger day (mm)	10.70 ± 2.18	11.17 ± 2.25	0.001
Number of retrieved oocytes	9.5(7,14)	13(10,16)	0.001
Number of fertilized oocytes	7(5,10)	9(7,12)	0.001
Number of D3 embryos	7(5,10.75)	9(7,12)	0.001
Number of quality D3 embryos	5(4,8)	7(5,10)	0.001
Number of blastocysts raised	2(2,5)	5(3,7)	0.001
Number of quality blastocysts	1(0,2)	2(1,4)	0.001
Number of frozen embryos	2(2,5)	4(2,6)	0.001
Embryo transfer situation (%)			
Quality embryo transfer	92.59(300/324)	93.04(1535/1639)	0.479
Non-high quality embryo transfer	7.41(24/324)	6.35(104/1639)	0.479
Normal fertilization (%)	75.5(64.29,85.71)	75(62.5,85.71)	0.223
Quality embryo (%)	83.33(71.43,1)	85(68.75,1)	0.292
Blastocyst rate	34.31(26.25, 52.71)	53.59(38.46, 71.43)	0.001
Quality blastocyst rate	36.36(0.00, 66.67)	50.00(33.33, 71.43)	0.001
Single embryo transfer (%)	33.64(109/324)	87.37(1432/1639)	0.001
Double embryo transfer (%)	66.36(215/324)	12.63(207/1639)	0.001

*LH *Luteinizing hormone, *E2* serum estradiol, *P* serum progestero

### Comparison of pregnancy outcomes

#### D4A and D5A groups at single embryo transfer

In single embryo transfer, we have performed 109 cycles of morula transfers and 1432 cycles of blastocyst transfers. The clinical pregnancy rate, miscarriage rate, live birth rate, preterm birth rate, overdue birth rate, twin birth rate, natural birth rate, cesarean delivery rate, neonatal birthweight, proportion of fetal macrosomia, proportion of low birthweight infants, and incidence of OHSS were not found to be significantly different between the D4A group and D5A group, as shown in Table 3.

**Table 3:** Comparison of pregnancy outcomes between the D4A and D5A groups at single embryo transfer

Variables	D4A group(*n* = 109)	D5A group(*n* = 1432)	*P* value
Clinical pregnancy (%)	59.63(65/109)	64.73(927/1432)	0.284
Miscarriage (%)	16.92(11/65)	13.05(121/927)	0.374
Live birth (%)	49.54(54/109)	56.28(806/1432)	0.346
Preterm birth (%)	1.83(2/109)	4.12(59/1432)	0.355
Overdue birth (%)	3.67(4/109)	3.63(52/1432)	1.000
Twin birth (%)	0(0/65)	1.83(17/927)	0.620
Natural birth (%)	42.59(23/54)	47.89(386/806)	0.450
Cesarean (%)	57.41(31/54)	52.11(420/806)	0.450
Newborn birthweight(g)	3209.56 ± 429.69	3260.10 ± 546.69	0.506
Fetal Macrosomia (%)	3.70(2/54)	5.98(49/820)	0.696
Low birthweight infant (%)	5.56(3/54)	7.44(61/820)	0.806
OHSS incidence (%)	0.92(1/109)	1.33(19/1432)	1.000

Unknown birthweight of D5A babies for 3 cycles

#### D4B group and the D5B group at double embryo transfer

In double embryo transfer, we have performed 215 cycles of morula transfers and 207 cycles of blastocyst transfers. There were no significant differences in clinical pregnancy rate, miscarriage rate, live birth rate, preterm birth rate, overdue birth rate, twin birth rate, natural birth rate, cesarean section rate, proportion of fetal macrosomia and OHSS incidence between the D4B and D5B group populations, but the birthweight of newborns in the D4B group was lower than that in the D5B group and the proportion of low birthweight infants was higher than that in the D5B group, as shown in Table 4.

**Table 4:** Comparison of pregnancy outcomes between the D4B and D5B groups at double embryo transfer

Variables	D4B group(*n* = 215)	D5B group(*n* = 207)	*P* value
Clinical pregnancy (%)	69.30(149/215)	65.22(135/207)	0.371
Miscarriage (%)	11.41(17/149)	8.15(11/135)	0.357
Live birth (%)	61.40(132/215)	59.90(124/207)	0.754
Preterm birth (%)	14.88(32/215)	13.04(27/207)	0.586
Overdue birth (%)	2.33(5/215)	2.90(6/207)	0.712
Twin birth (%)	36.24(54/149)	34.81(47/135)	0.802
Natural birth (%)	31.06(41/132)	27.42(34/124)	0.522
Cesarean (%)	68.94(91/132)	72.58(90/124)	0.522
Newborn birthweight(g)	2777.33 ± 652.96	2915.59 ± 602.46	0.039
Fetal Macrosomia (%)	3.23(6/186)	2.34(4/171)	0.823
Low birthweight infant (%)	34.95(65/186)	21.05(36/171)	0.004
OHSS incidence (%)	1.86(4/215)	1.45(3/207)	1.000

#### Single and double embryo transfer in group D4

**Fig. 3:**
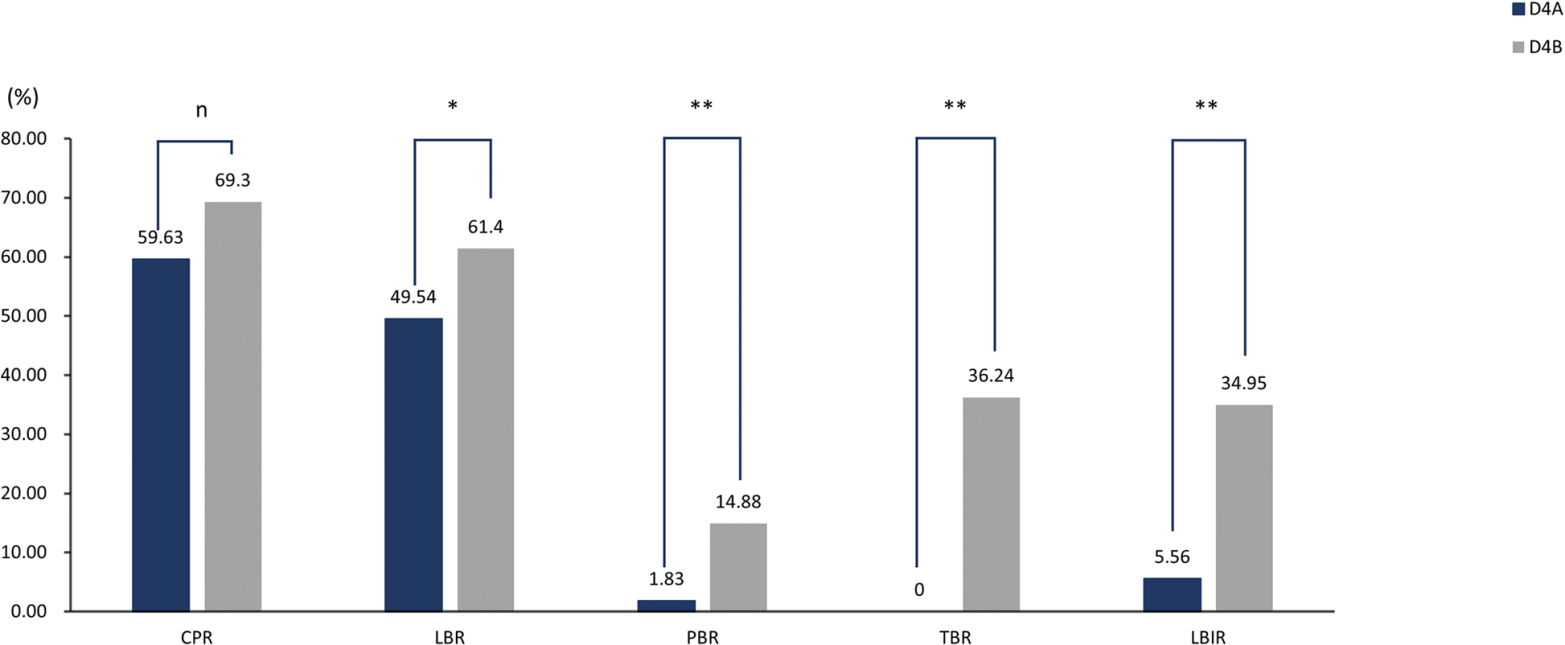
Comparison of single and double embryo transfer assisted pregnancy outcomes in the D4 group. D4A, single morula was transplanted; D4B, double morulae were transplanted; CPR, Clinical pregnancy rate; LBR, Live birth rate; PBR, Preterm birth rate; TBR, Twin birth rate; LBIR, Low birthweight infant rate; np> 0.05, * *p* < 0.05, ** *p* < 0.01

Among 324 D4 morula transfer cycles, we transferred one embryo in 109 cycles and two embryos in 215 cycles. There were no significant differences in clinical pregnancy rate, miscarriage rate, overdue delivery rate, natural delivery rate, cesarean delivery rate, proportion of fetal macrosomia and OHSS incidence between patients in D4A and D4B groups, but the live birth rate, preterm delivery rate, twin birth rate and proportion of low birthweight infants were lower in D4A group than in D4B group; neonatal birthweight was higher than in D4B group, as shown in Table 5; Fig. 3.

**Table 5:** Comparison of single and double embryo transfer assisted pregnancy outcomes in the D4 group

Variables	D4A group(*n* = 109)	D4B group(*n* = 215)	*P* value
Clinical pregnancy (%)	59.63(65/109)	69.30(149/215)	0.082
Miscarriage (%)	16.92(11/65)	11.41(17/149)	0.271
Live birth (%)	49.54(54/109)	61.40(132/215)	0.037
Preterm birth (%)	1.83(2/109)	14.88(32/215)	0.001
Overdue birth (%)	3.67(4/109)	2.33(5/215)	0.735
Twin birth (%)	0(0/65)	36.24(54/149)	0.001
Natural birth (%)	42.59(23/54)	31.06(41/132)	0.133
Cesarean (%)	57.41(31/54)	68.94(91/132)	0.133
Newborn birthweight(g)	3209.56 ± 429.69	2777.33 ± 652.96	0.001
Fetal Macrosomia (%)	3.70(2/54)	3.23(6/186)	1.000
Low birthweight infant (%)	5.56(3/54)	34.95(65/186)	0.001
OHSS incidence (%)	0.92(1/109)	1.86(4/215)	0.667

#### Single and double embryo transfer in group D5

**Fig. 4:**
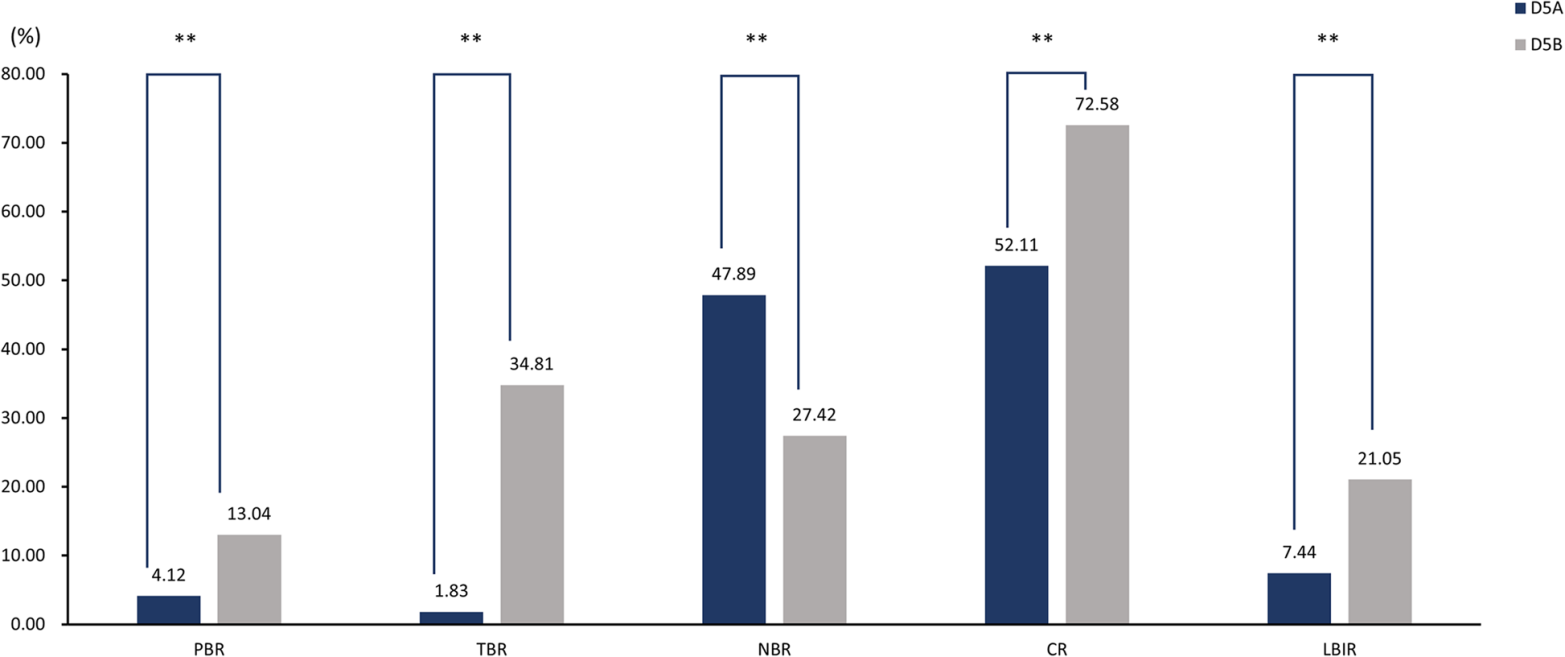
Comparison of single and double embryo transfer assisted pregnancy outcomes in the D5 group. D5A, single blastocyst was transplanted; D5B, double blastocysts were transplanted. PBR, Preterm birth rate; TBR, Twin birth rate; NBR, Natural birth rate; CR, Cesarean rate; NB, Newborn birthweight; LBIR, Low birthweight infant rate; ** *p* < 0.01

Among 1639 D5 blastocyst transfer cycles, we transferred one embryo in 1432 cycles and two embryos in 207 cycles. There were no significant differences in clinical pregnancy rate, miscarriage rate, live birth rate, overdue birth rate, proportion of fetal macrosomia, or incidence of OHSS between patients in the D5A and D5B groups. However, the preterm birth rate, twin birth rate, cesarean section rate, and proportion of low birthweight infants were lower in the D5A group; the natural birth rate and neonatal birthweight were higher in the D5A group, as shown in Table 6; Fig. 4.

**Table 6:** Comparison of single and double embryo transfer assisted pregnancy outcomes in the D5 group

Variables	D5A group(*n* = 1432)	D5B group(*n* = 207)	*P* value
Clinical pregnancy (%)	64.73(927/1432)	65.22(135/207)	0.892
Miscarriage (%)	13.05(121/927)	8.15(11/135)	0.107
Live birth (%)	56.28(806/1432)	59.90(124/207)	0.326
Preterm birth (%)	4.12(59/1432)	13.04(27/207)	0.001
Overdue birth (%)	3.63(52/1432)	2.90(6/207)	0.594
Twin birth (%)	1.83(17/927)	34.81(47/135)	0.001
Natural birth (%)	47.89(386/806)	27.42(34/124)	0.001
Cesarean (%)	52.11(420/806)	72.58(90/124)	0.001
Newborn birthweight(g)	3260.10 ± 546.69	2915.59 ± 602.46	0.007
Fetal Macrosomia (%)	5.98(49/820)	2.34(4/171)	0.055
Low birthweight infant (%)	7.44(61/820)	21.05(36/171)	0.001
OHSS incidence (%)	1.33(19/1432)	1.45(3/207)	1.000

Unknown birthweight of D5A babies for 3 cycles

### Comparison of live birth outcomes

#### Factors affecting live birth

There was no significant difference in BMI, AMH, infertility years, D4 or D5 embryo transfer, single or double embryo transfer between the live birth group and the non-live birth group. However, the age of the live birth group was younger than that of the non-live birth group, and the endometrial thickness was greater than that of the non-live birth group, as shown in Table 7.

**Table 7:** Factors affecting live births

**Group**	**Cycles**	**Age(years)**			**BMI(kg/m2)**			**AMH(ng/mL)**			**Em (mm)**
Live birth	1116	30.78 ± 4.19			21.96 ± 3.09			4.48 ± 8.29			11.2 ± 2.21
Non-live birth	847	31.77 ± 4.38			21.90 ± 3.19			4.18 ± 3.42			10.95 ± 2.29
t		5.05			0.36			0.66			2.45
***P***		0.001			0.718			0.508			0.014
**Group**	**DIFY**	**Embryo days transferred**					**Number of embryos transferred**				
		**D4(%)**		**D5(%)**			**Single(%)**			**Double(%)**	
Live birth	3(2, 4)	16.67 (186/1116)		83.33 (930/1116)			77.06 (860/1116)			22.94 (256/1116)	
Non-live birth	3(2, 5)	16.29 (138/847)		83.71 (709/847)			80.40 (681/847)			19.60 (166/847)	
t	0.50	0.049		0.049			3.184			3.184	
***P***	0.61	0.825		0.825			0.074			0.074	

*Em *Endometrial thickness on trigger day, *DIFY *Duration of Infertility in Years

#### Logistic regression analysis of factors related to live birth rate

Live birth outcome was used as the dependent variable (live birth = 1, not live birth = 0), and the influential factors that differed (age, endometrial thickness at hCG triggering) as well as the transfer of D4 or D5 embryos, single or double embryo transfers were included in the analysis of the independent variables into the model for a binary logistic regression analysis, which showed that age was a risk factor for the outcome of live births (OR = 0.945, 95% CI: 0.9250.965, *P*<0.05), endometrial thickness at hCG triggering and number of embryos transferred were protective factors for live birth outcome (OR = 1.05, 95% CI: 1.081.093; OR = 1.351, 95% CI:1.079 ~ 1.691, *P*<0.05). while D4 or D5 embryo transfers were not associated with live birth outcomes (*P*>0.05). as shown in Table 8.

**Table 8:** Logistic regression analysis of factors related to live birth rate

Variable	B	SE	Wald χ2	*P* value	OR (95%CI)
Age(years)	-0.057	0.01	27.21	0.001	0.945 (0.925, 0.965)
Em (mm)	0.048	0.02	5.48	0.019	1.05 (1.008, 1.093)
Embryo days transferred	0.13	0.14	0.83	0.361	1.139 (0.861, 1.507)
Number of embryos transferred	0.3	0.12	6.86	0.009	1.351 (1.079, 1.691)

*B *Regression Coefficient, *SE *Standard Error, *Em *Endometrial thickness on trigger day

#### ROC curves for impact factors related to live birth rate

**Fig. 5:**
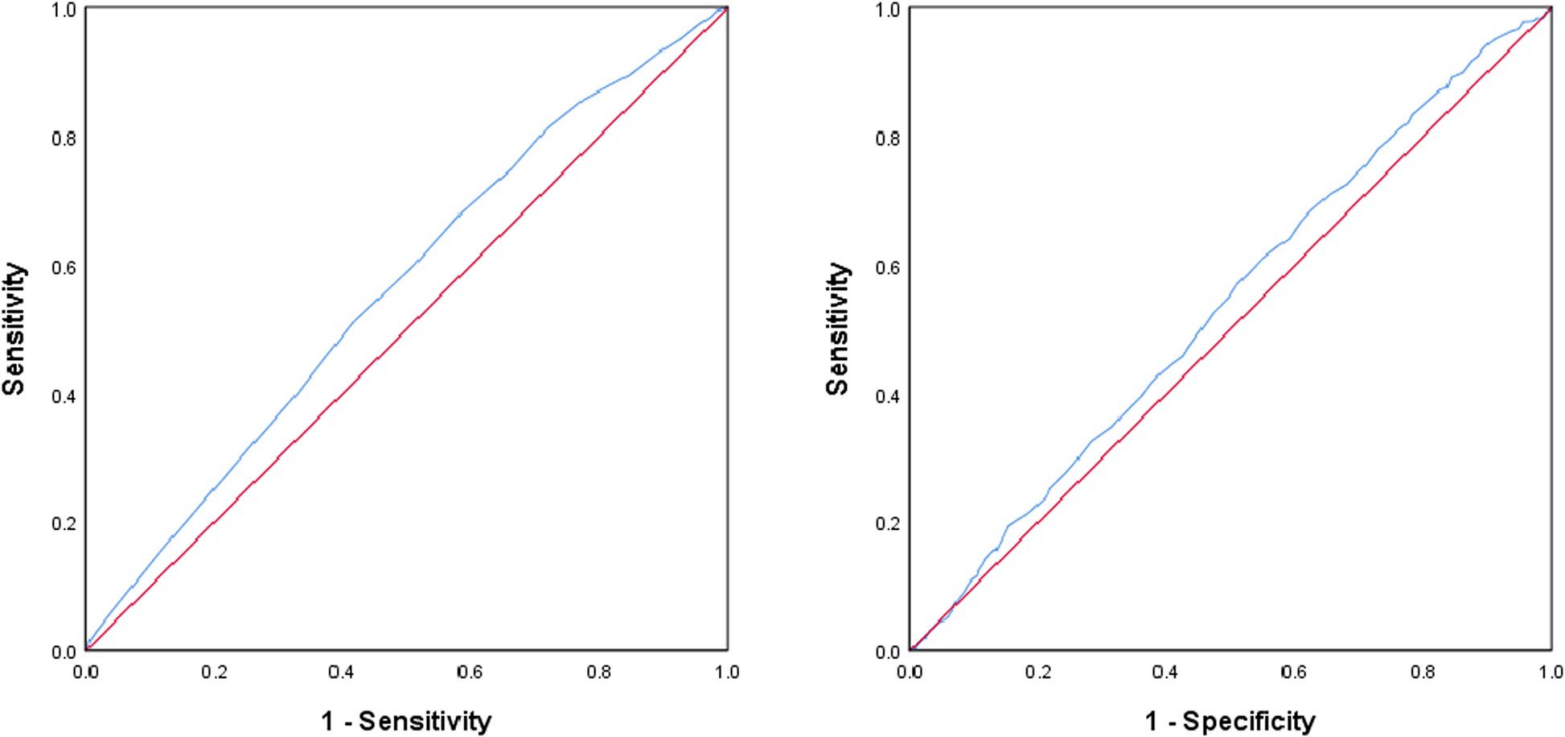
**a** ROC curve of Age. **b** ROC curve of Endometrial thickness

The ROC curve for age has an AUC of 0.563, with an ideal cutoff value of 32.5 years, a Yoden index of 0.096, a sensitivity of 41.40%, and a specificity of 68.20%. The AUC for endometrial thickness is 0.537, with an ideal cutoff value of 10.45 mm, a Yoden index of 0.061, a sensitivity of 61.10%, and a specificity of 45.00%. See Fig. 5a and b, and Table 9.

**Table 9:** ROC curves for impact factors related to live birth rate

Variable	AUC	Cut Off	*P* value	95%CI	
				lower limit	upper limit
Age(years)	0.563	32.50	0.001	0.538	0.589
Em (mm)	0.537	10.45	0.005	0.511	0.563

*Em *Endometrial thickness on trigger day

## Discussion

The selection of embryos with optimal implantation potential for transfer is a critical step in optimizing pregnancy outcomes in vitro fertilization-embryo transfer (IVF-ET) treatment [21]. Studies have shown that embryo culture is a meritocratic process and that a large number of embryonic genes are activated and expressed on day 4 compared to day 3 transfer, indicating that D4 morula development is an essential screen for future embryo survival [8, 22, 23]. Sang et al. examined morula and maternal endometrium transcriptome datasets to identify the expression of receptor genes expressed by morula and corresponding ligand genes in endometrium, and to further clarify the details of communication between embryo and mother [24].Human embryos enter the uterine cavity on day 4 after fertilization, when the uterus provides a different nutritional environment than the oviduct, uterine contractility is reduced, and synchronization of embryos with the endometrium is improved [25], giving D4 morula and D5 blastocyst transfers an advantage over D3 cleavage embryo. Two main morphogenetic phases build the basic architecture of the pre-implantation embryo: the formation of the morula and of the blastocyst [26]. Embryo culture up to day 5 may aid in the rejection of unsuitable embryos, but it is unknown whether D5 blastocyst is superior to D4 morula in this regard. However, compared to D5 blastocyst, transfer of D4 morula reduces the exposure time of embryos in vitro, reduces the impact of environmental factors on embryos in vitro [27, 28], reduces the risk of developmental arrest and embryo quality decline [29], and makes better use of embryos. Patients undergoing blastocyst culture may have fewer total embryos available for transfer, or no embryos available for transfer and transfer cycles cancelled. However, it should be noted that with the development of time-lapse camera technology, it is possible to integrate optical microscopy systems with incubators, capture images of embryos at set time intervals, generate videos by overlaying the timeline in order to dynamically observe the development of the embryo, intelligently calculate the cellular activity of the embryo, illustrate the phenomenon of cell division, and efficiently analyze the data of the embryo to select the optimal mulberry or blastocyst embryo for transplantation [30]. . Furthermore, unlike traditional incubators, the time-delay system avoids frequent switching on and off of the incubator and keeps the environment stable, particularly the pH and temperature, which reduces the risk of fertilized eggs in the process of observing and moving and the influence of environmental factors on the embryos [31]. Overall, combining the advantages and risks, the outcome of transferring D4 morula versus D5 blastocyst has not been clearly established.

The results of this study show that there are no significant differences in general baseline conditions such as age and BMI between the D4 and D5 groups, but the basic E2, E2 and endometrial thickness at hCG triggering, of retrieved oocytes, number of fertilized oocytes, number of D3 embryos, number of quality D3 embryos, number of blastocysts raised, number of quality blastocysts, number of frozen embryos, blastocyst formation rate, and quality blastocyst rate, and percentage of single embryo transfer are lower in the D4 group than in the D5 group, while the percentage of double embryo transfer is higher than in the D5 group. Based on basic laboratory and embryo data, it was easy to find that despite the fact that D3 quality embryos with a number of embryos greater than or equal to 3 were selected for culture and the best possible morulae or blastocysts were selected for transfer (D4 and D5 excellent embryo transfer rate of 92.95% vs. 93.04%, *P* < 0.05), the basic condition of the patients and the condition of the embryos in the D5 group were still better than those in the D4 group. This could be related to our transfer strategy, in which we choose single blastocyst transfer as the primary strategy when the overall circumstances are favorable and the blastocyst-raising success rate is high. In contrast, we preferentially transfer one or two D4 morulae when there are few embryos, an anticipated delay in embryo growth on day 4, a risk of cycle cancellation, or a conflict in schedule for day 5 transfer. Therefore, the clinical and embryonic profile of the D5 group may be slightly better than that of the D4 group, while the single embryo transfer rate is significantly higher than that of the D4 group. However, despite the less favorable clinical and embryonic conditions in the D4 group than in the D5 group, further comparisons of pregnancy outcomes in this study revealed similar pregnancy outcomes in the D4 and D5 groups, which was consistent with previous studies.

Kang et al. performed a retrospective analysis of 271 cycles and discovered no significant changes in clinical pregnancy, live birth, or miscarriage rates between D4 and D5 single embryo transfers, despite the fact that the miscarriage rate was somewhat higher in D4. They suggested that the results may have been affected by sample size constraints and that additional samples are required for validation [32]. In a retrospective study of 427 GnRH antagonist protocol cycles, Li et al. found no significant difference in clinical pregnancy rate, live birth rate, or miscarriage rate between D4 and D5, however the full-term birth rate was greater in the D4 group than in the D5 group. They also presented an embryo quality assessment system for D4 to help with embryo selection [33]. Simopoulo et al. reviewed 6 prospective studies and 9 retrospective cohort studies, and found that the rates of clinical pregnancy, sustained pregnancy/live birth, cancelled pregnancy, and miscarriage of D4 and D5 transplantation had no statistical difference. Additionally, statistically speaking, D4 had a much lower preterm birth rate than D5 [21].

In this study, we conducted a retrospective analysis of 1963 cycles using an early follicular phase ultra long protocol. Due to the significant difference in the number of embryo transfers between D4 and D5 transfers (D4 and D5 single embryo transfer rates of 33.64% vs. 87.37%), in order to exclude the impact of embryo transfer numbers on pregnancy outcomes, this study further stratified the analysis of single and double embryo transfers, finding that there was no significant difference in D4 transfer and D5 transfer outcomes for single embryo transfer, and the results were generally consistent with the previous studies. Moreover, there was no significant difference in the primary pregnancy outcome between D4 and D5 double embryo transfers. Although, the birthweight of babies in the D4B group was lower than that of babies in the D5B group(2777.33 ± 652.96 vs. 2915.59 ± 602.46, *P*<0.05), and the low birthweight infant was higher in the D4B group than in the D5B group(34.95% vs. 21.05%, *P*<0.05). The weight of newborns is an important indicator of children’s growth and development, with a normal range of 2500–4000 g. Doron et al. discovered a link between embryonic morphodynamic parameters and the occurrence of low birth weight infants [34]. In comparison to the D5 blastocyst, the D4 morula has undergone a briefer duration of in vitro culture, resulting in incomplete development. Consequently, their cell division and growth might not reach optimal levels, thus limiting their growth potential to some degree. However, there was no significant difference in the proportion of newborn birth weight and low birth weight infants between D4 and D5 embryo transfers in the previous text (3209.56 ± 429.69 vs. 3260.10 ± 546.69, 5.56% vs. 7.44, *P* > 0.05). This may be due to the fact that the subtle differences in growth and development potential are relatively easy to overlook during a single pregnancy. However, when transferring two embryos, the twin rate increases, and the requirements for embryonic development potential become more stringent. Therefore, compared to transplanting two D5 blastocysts, the growth and development of embryos may be more susceptible to adverse effects when transplanting two D4 morulae. Certainly, there are many factors that affect the birth weight of newborns, including the health status, nutritional intake, and lifestyle of the mother [35]. Due to the small sample size of twin transplants in this study, more research are needed to determine if morula embryo transplantation during twin pregnancies impacts fetal growth and development.

In order to find the optimal transfer strategy, we compared the outcomes of single and double embryo transfer on days D4 and D5, respectively, and found that the live birth rate, preterm birth rate, twin birth rate, and low birthweight infant percentage were lower in the D4A group than in the D4B group at D4 transfer(49.54%vs61.40%、1.83%vs14.88%、0%vs36.24%、5.56%vs34.95%, *P*<0.05), while the newborn birthweight was higher than in the D4B group(3209.56 ± 429.69vs2777.33 ± 652.96, *P*<0.05). In addition, the clinical pregnancy rate in the D4A group was lower than that in the D4B group(59.63%%vs69.30%,*P* = 0.082), and the natural birth rate was higher than that in the D4B group(42.59%vs31.06%,*P* = 0.133). Although the differences were not statistically significant, they still showed a corresponding trend, which may be related to the small sample size of D4 transplants in this study, and further increasing the sample size may make the above differences significant. The results of our study suggest that the outcome of double embryo transfer in D4 will be better than single embryo transfer in terms of success rate. According to Duffy et al., twin pregnancies lead to the occurrence of outcomes such as preterm delivery, cesarean delivery, and low birthweight infants [10], thus, we primarily blamed the difference in the twin pregnancy rate for the difference in secondary pregnancy outcomes between the D4A and D4B groups. In our study, double embryo transfer in D4 row significantly increased the twin rate and increased the risk of pregnancy such as preterm delivery and low birthweight. Several studies have shown that reducing the number of embryos transferred helps to reduce the occurrence of multiple pregnancies as well as maternal and infant adverse pregnancy outcomes [9, 36], and we also tend to control the number of embryos transferred in our transfer strategy and promote single embryo transfer to reduce the risk of twin pregnancies. In the D5 transfer, there was no significant difference in the main pregnancy outcomes between single and twin embryo transfer, and the preterm delivery rate, twin birth rate, cesarean delivery rate, and low birthweight infant ratio in the D5A group were lower than those in the D5B group, while the natural delivery rate and neonatal birthweight were higher than those in the D5B group, indicating that single embryo transfer would be more advantageous in the D5 transfer.

Additionally, we found a higher rate of cesarean delivery in our study for both D4 and D5. The reasons for this are diverse. On the one hand, the increased rate of multiple births in assisted reproduction increases the probability of difficult deliveries [23, 37]. On the other hand, the relatively advanced maternal age [37], the mental stress of late pregnancy, and the difficulty in obtaining a fetus result in the request for cesarean delivery by the mother and her family. In response, the indications for cesarean delivery were relaxed clinically, which led to an increase in the rate of cesarean delivery. Besides, as an important complication of IVF, OHSS syndrome was characterized by increased vascular permeability, clinical manifestations such as ascites, oliguria, and even life-threatening respiratory failure. An increase in the number of embryos obtained increases the risk of OHSS [38]. Xin et al. found that when patients at high risk for OHSS underwent embryo transfer, the incidence of OHSS was lower in the D5 transfer group than in the D3 group. They hypothesized that this may be because the delayed culture gave doctors more time to monitor and treat patients’ symptoms as well as the lower rate of multiple births with D5 transfer [39].However, no studies have found a link between D4 or D5 transfer and OHSS. In our study, no significant differences were seen in the incidence of OHSS under different transplantation strategies, which may be due to the limitations of the retrospective study model we adopted and the inadequate sample size, and further studies are needed to explore the association in the future.

Many factors affect pregnancy outcomes in IVF-ET, a review and meta-analysis by Vitagliano et al. found that embryonic aneuploidy variants associated with maternal age were considered to be a significant limiting factor in pregnancy outcomes, and that an increase in maternal age was associated with a decrease in ART success rates [40]. Sermondade et al. reviewed that BMI is an important factor influencing assisted reproduction outcomes, and that female obesity has a significant negative impact on live birth rate after in vitro fertilization [41]. In addition, factors such as decreased ovarian function and thin endometrium can also lead to adverse pregnancy outcomes and birth defects [42, 43]. Except for endometrial thickness, there were no significant differences in patient characteristics such as age, BMI, and AMH between groups D4 and D5 in this study. In order to further explore the factors affecting pregnancy outcome, to exclude the role of confounding factors on pregnancy outcome, and to further validate the effects of D4 morula and D5 blastocyst transfer on live births, the present study was conducted as a one-way analysis with the outcome of live birth as the dependent variable. It was found that the live birth group was younger than the non-live birth group (30.78 ± 4.19 vs. 31.77 ± 4.38, *P* < 0.05), and the endometrial thickness was thicker on the day of hCG (11.2 ± 2.21 vs. 10.95 ± 2.29, *P* < 0.05), whereas there was no significant difference in the number of embryos or days transferred. The research found that the live birth group was younger than the non-live birth group (30.78 ± 4.19 vs. 31.77 ± 4.38, *P*<0.05) and had thicker endometrium (11.2 ± 2.21 vs. 10.95 ± 2.29, *P*<0.05). However, there were no significant differences between the transplantation of morula or blastocyst, or the transplantation of single or double embryos. After controlling for confounding factors, logistic regression analysis showed age as a risk factor for live birth outcomes (OR = 0.945, 95% CI: 0.925 ~ 0.965, *P* < 0.05) and endometrial thickness as a protective factor (OR = 1.05, 95% CI: 1.008 ~ 1.093, *P* < 0.05), supporting previous studies. We also found that the number of embryos transferred was also a protective factor for live birth outcome (OR = 1.351, 95% CI: 1.079–1.691, *P* < 0.05), indicating that patients undergoing double embryo transfer have a higher live birth rate than single embryo transfer. However, there was no significant difference in live birth outcomes between D4 transplantation and D5 transplantation (*P*>0.05), indicating that D4 morula transfer had a similar pregnancy outcome to D5 blastocyst transfer.

However, there were certain limitations to this study. Although we compared the differences in basal hormones, hormones after adjustment, and hormones on the day of HCG injection, we overlooked the collection and comparison of hormones on the day of embryo transfer. For instance, many studies have shown that excessively low or high serum progesterone levels on the day of transfer can affect the final outcome of ART [44, 45]. Therefore, a more comprehensive comparison and analysis was needed in this regard. Furthermore, the sample size of D4 in this study was relatively small, and the study didn’t analyze the quality and grading of embryos on D4 and D5 days, and didn’t compare pregnancy complications and subsequent neonatal mental and physical development. Further prospective randomized controlled studies are needed to verify the benefits of the above transfer strategies.

## Conclusion

Our findings suggest that D4 morula embryo transfer does not reduce IVF success in the early follicular phase extra-long protocol. The D4 embryo transfer can be a good alternative to D5 blastocyst transfer in case of holiday or work schedule conflicts. For D4 morula embryo transfer, single and double embryo transfer should be selected individually considering the success rate and the risk of twin pregnancy, while single blastocyst transfer is more recommended for D5 blastocyst transfer to reduce the rate of double pregnancy and optimize pregnancy outcome. Simultaneously, to improve pregnancy outcomes, it is critical to thoroughly examine individual parameters such as the patient’s age and endometrial thickness, and personalize the transplantation plan to each individual.

### Supplementary Information

Supplementary Material 1.

## Data Availability

Data is provided within the manuscript or supplementary information files.

## Abbreviations

D4: Day 4
D5: Day 5
IVF/ICSI: In vitro fertilization/intracytoplasmic sperm injection
ART: Assisted Reproductive Technology
D3: Day 3
GnRH-a: Gonadotropin releasing hormone agonist
FSH: Follicle stimulating hormone
LH: Luteinizing hormone
E2: Estradiol
Gn: Gonadotropin
hCG: Human chorionic gonadotropin
OHSS: Ovarian hyperstimulation syndrome
BMI: Body mass index
IVF-ET: in vitro fertilization-embryo transfer
PCOS: Polycystic ovary syndrome
